# A Peculiar Form of Iritis Characterized by a Gelatinous Mass in Anterior Chamber, at Times Resembling a Cataractous Lens

**Published:** 1896-04

**Authors:** S. Latimer Phillips

**Affiliations:** Savannah, Ga.


### A PECULIAR FORM OF IRITIS CHARACTERIZED BY A GELATINOUS MASS IN ANTERIOR CHAMBER, AT TIMES RESEMBLING A CATARACTOUS LENS.

BY S. LATIMER PHILLIPS, M.D.,

SAVANNAH, GA.

In 1871 Dr. Schmidt called attention for the first time to a form of iritis accompanied by an exudation into the anterior chamber and especially to that which resembled a dislocated cataractous lens, having been forced into the chamber.*

Gunning soon followed with three cases of free gray exudation into anterior chamber, calling it gelatinous, and saying that it was poured out suddenly and rapidly disappeared. After a few days no trace was left. Two of the cases were certainly caused by syphilis, while in the third it could not at least be excluded.†

Dr. E. Gruening, of New York, reported a case under the title of "Spongy Exudation in the Anterior Chamber."‡

He had seen this exudation before in the anterior chamber after operations upon the iris. He says Knapp characterizes this exudation as spongy. About twenty-four hours after an operation a brownish, shapeless mass is noticed, composed of filaments running in various directions and forming a network, resembling the gross structural arrangements of a sponge. The spongy exudation either disappears in this form or is transformed into a bluish-white homogeneous and sharply defined mass before doing so. Gunning's case was one in which the exudation came on from a gummy iritis, the sequel of syphilis contracted two years before. It was poured out rapidly into the anterior chamber, and on the fifth day had completly disappeared.

C. J. Kipp|| reports a case in which the iritis appeared four months after the inoculation of syphilis, and the eruption was still on forehead, with mucous patches in mouth. He noticed a slight point of elevation in the iris near its pupillary margin. The next day it

*Eigen Thümlich geformte Exsudate bei Iritis. Klin. Monats. für Augenheilk, s. 94.

†Klin. Monats. für Augenheilk, B'd x., s. 7.

‡Arch. of Ophthal. and Otology, vol. iii.

||Arch. of Ophthal. and Otology, vol. iii., No 1.

was covered by a grayish exudation, and on the fourth day the anterior chamber was almost completely filled with a semi-transparent grayish mass. Sixteen days after the first appearance of the tumor all the exudation had disppeared, the point of the condyloma only being indicated by a yellowish discoloration of the iris and a posterior synechia.

Alexander* saw altogether six of these cases in his practice, and in every one of them syphilis was undoubtedly the cause. The first of these cases he saw in 1867, in a syphilitic patient with repeatedly recurring plastic iritis. In the anterior chamber, at the margin of the lower border of the pupil, with clearly defined outlines, was a gray opaque exudation; with every motion of the head there was a tremulous movement inward. After eight days it had entirely disappeared. Of the remaining cases, two had exudation resembling dislocated lenses. The lower half of the anterior chamber was filled with the mass, only the border of the iris showing All of these cases had the usual treatment for syphilis, and complete absorption took place. Alexander thinks, though it may be possible from other causes, such as trauma, that syphilis is the chief one.

C. J. Kipp† has seen cases of gonorrhœic irido-choroiditis in which there was exudation in the anterior chamber, upon coagulation, resembling an opaque lens, suspended in front of the pupil. This was always absorbed, and in most of the cases the vision was completely restored. A few though showed impaired vision from membranes, etc.

Dr. Ernest Fuchs,‡ in speaking of this form of exudation, says it is found in every kind of acute iritis occasionally. It rapidly skrinks, in a few days being entirely gone, or leaving only a thin membrane, and sometimes a small thread attached to the edge of the pupil.

The histology, as given by Adolph Alt,|| is that there are numerous large and small hemorrhages into the parenchyma of the iris. The fluid portions of the blood are transuded into the anterior

*Syph. und Auge, s. 70.

†New York Record, vol. 38, No. 2, p. 40.

‡Lehrbuch der Augenhielkunde, 1889, s. 302.

||Lectures on the Human Eye, 1884, p. 86.

chamber, while its cellular elements remain in the iris tissue and undergo fatty degeneration. The fibrin of the blood coagulates in the anterior chamber, but is later on dissolved. Sometimes two kinds of exudation are found in the anterior chamber, a gelatinous and a sero-fibrinous. The latter, under a microscope, is found to be a network of fine fibrinous threads, like those found in the alveoli of croupous pneumonia, and is filled with serum and a small number of lymph cells. The former is a uniform transparent substance, differing from the aqueous humour only in being gelatinous. The absorption of this exudation always begins nearest the cornea and progresses toward the center. Alt designates this as a variety of serous iritis.

Having met with two cases in my practice which would answer to the clinical description above, I report them :

I. Henry W., a large, well-formed negro man, of thirty-three years, presented himself for treatment with the following history: Has had repeated attacks of inflammation of eyes (recurrent iritis) and sight slowly getting worse until now, when he can scarcely see at all. R. counts fingers at two feet; L. only has light appreciation. His attacks were always accompanied by severe pains in head and eyes. At the time has but little in right. Slight peri-corneal injection. Pupil of left eye closed by membrane, sequel of old iritis. No irritation about this eye now. R. eye, find lower half of anterior chamber filled with a dull, grayish opaque mass. Under atropia pupil dilates upward, leaving a small free opening. The mass seems to have its origin at the lower and outer margin of the pupillary border, and pushing out seems suspended before the pupil. The iris which shows above is of a dingy, muddy color, and seems somewhat pressed forward where the exudation has its origin. I could get no clear history of syphilis in this case, though he confessed to having had buboes eighteen years before. Notwithstanding, he was put on gr. $\frac{1}{2}$ bichloride t. i. d., to be increased. The eye was treated with atropia, and closed by a compress and bandage. He was under my treatment for three weeks; at the end of that he disappeared, and I have not seen him since. When I last saw him the exudation had much diminished in size, and vision was some better.

II. Brown Weathers, colored, aged nineteen. Two months since contracted syphilis in the usual way. Now has scaly eruptions over both upper and lower limbs, with enlarged inguinal glands and mucous patch in mouth. Lips thick and hanging, with discharge from nose, tendency to skin ulceration at alæ. Altogether in a badly nourished state. R. eye v. $= \frac{20}{xx}$. L. v. $= \frac{20}{cc}$. Was taken with severe pain in left eye two days ago, it being always more severe at night. Eye red and photophobic; in fact has all symptoms of iritis. Pupil responds to light but sluggishly. In anterior chamber is a light, pearl gray mass, partially filling it. Apparently it has sprung out from the inner pupillary margin of the iris, and extends in toward the periphery. It juts out some into the pupil, but does not extend clear across. The outlines of the mass are clearly defined, and at the point in the iris where the exudation seems to have its origin, the iris does not respond to atropia, indicating a growth there, or some plastic exudation binding the iris to the anterior capsule of lens. The mass projects out into the anterior chamber, and at its most prominent point almost comes in contact with Descemet's membrane. There is no movement of the mass synchronous with that of the head. Tension normal. Atropia locally, with compress and bandage. He was put upon gr. $\frac{1}{16}$ hyd. bichlor. t. i. d., with gr. x of pot. iodid. t. i. d.

Three days later the gray body diminishing rapidly. Four days after this very much shrunken, and looks like a thin membrane of slightly brownish hue now. Long axis has become vertical. A few small spots are seen on capsule now, where it has come in view; pupil well dilated.

Ten days later, nine days after I saw case, all appearance of the mass gone, save a yellowish brown point size of a pin head, in the iris near its pupillary margin; vision nearly normal. No redness about eye. I did not see patient again after this.

Here we have two cases of iritis, one positively due to syphilis, the other most probably, with a peculiar, outpouring grayish matter into the anterior chamber. What is this, and where does it come from?

There are two distinct forms of syphilitic iritis—the plastic or papular, occurring at the same time with secondary manifestations of the disease, as eruptions over skin, mucous patches, etc. Here, no doubt, the same thing takes place as does in the skin, papular eruption, but being modified by the anatomical structure of the iris. It is from these papules that the exudation springs, and, as in other cases of plastic iritis the exudation is absorbed. Serous iritis never occurs in syphilitics as a manifestation of the disease. The other form being gummy, takes place in the late stages of syphilis. The gummy tumor varies from the size of a pea to that of half a hazelnut, and generally has its location in the region of the ciliary border of the iris. It is not absorbed, but undergoes fatty degeneration, leaving a scar in the iris tissues.

